# Writing Increases Precision-Weighted Inference of Conceptual Organization: A Bayesian-Brain Active-Inference Model of the Writing-over-Speaking Advantage in Analytic Thinking

**DOI:** 10.3390/brainsci16080771

**Published:** 2026-07-23

**Authors:** Angelica Maria Silva, Renata Melanie Truelove, Anthony Millán De Lange, Roberto Limongi

**Affiliations:** 1 Department of Francophone Studies and Languages, Faculty of Arts, Brandon University, 270—18th Street, Brandon, MB R7A 6A9, Canada; silvaa@brandonu.ca; 2 St. Stephen’s College, University of Alberta, Edmonton, AB T6G 2J6, Canada; rtruelov@ualberta.ca; 3 Department of Psychology, School of Psychology, Faculty of Humanities, Arts, and Social Sciences, Universidad del Norte, Km. 5 vía Puerto Colombia, Barranquilla 081007, Colombia; delangea@uninorte.edu.co; 4 Department of Psychology, Faculty of Science, Brandon University, 270—18th Street, Brandon, MB R7A 6A9, Canada

**Keywords:** active inference, natural language processing, analytic thinking, writing

## Abstract

**Highlights:**

**What are the main findings?**

- Under laboratory-controlled conditions, university students produced written responses with higher NLP-derived analytic thinking scores than spoken responses.
- Relative to speaking cues, writing cues increased the active-inference agent’s probability of occupying an active state corresponding to high-analytic-thinking discourse production.

**What are the implications of the main findings?**

- Computational phenotypes estimated from writing samples could support future research on language-production markers of psychological disorders.
- Active-inference models of analytic thinking could be extended and validated in populations of individuals with psychological disorders under laboratory-controlled conditions.

**Abstract:**

Background: The analytic thinking score (ATS) has been interpreted as a linguistic marker of organized thought. Written language typically shows a higher ATS than spoken language. From the perspective of active inference under the free-energy principle, we proposed a preliminary neurocomputational model of this writing-over-speaking advantage. We propose that ATS is an externally computed linguistic measure reflecting analytic-thinking active states that arise from precision-weighted inference over internal conceptual-organization (CO) states. We hypothesize that written production shows a higher ATS when the active-inference agent increases posterior confidence in high-CO states. Methods: ATSs were extracted from written and spoken samples produced by university students who described thematic apperception test images. Participants were modeled as active-inference agents using a two-timestep Markov decision process (MDP) in which speaking and writing sensory cues updated beliefs about internal CO states which then drove analytic thinking active states. Belief updating was formalized through marginal message-passing and theoretically interpreted in terms of prediction-error signaling and precision-weighted neuronal synaptic gain. An attention-related parameter (AP) controlled the precision of the CO-sensory state mapping. Bayesian model selection was used to assess the model’s preliminary construct validity. Results: Written responses showed higher ATSs than spoken responses. The AP estimate indicated that writing cues supported posterior inference toward high-CO states stronger than speaking cues. Bayesian model selection favored the active-inference MDP over a Variational Laplace linear model. Conclusions: The current preliminary evidence speaks to a candidate active-inference model in which writing ascribes higher precision-weighted inference of CO, reflected in higher ATSs.

## 1. Introduction

Language serves as a fundamental tool for organizing and expressing thoughts. It not only enables communication but also reflects underlying cognitive processes that guide how individuals interpret, organize, and interact with the world [1]. Consider, for example, a picture-description task, such as the thematic apperception test [2]. In this task, participants generally produce short narratives about a visual depiction, for example a picture or drawing. The basic trial consists of two stages. First, the participant expects the presentation of the visual depiction. Second, once the visual depiction is shown, the participant produces a short narrative about its content. 

Narrative production entails at least two stages: prelinguistic and linguistic. In the prelinguistic stage, the speaker constructs an internal representation of the picture content whereas in the linguistic stage the speaker translates or encodes that representation into words [3,4,5,6,7]. Crucially, these stages are not unique to speaking. The analog in writing-production models is the planning, idea-generation, or content-generation stage, in which writers generate, select, and organize ideas before translating them into written language [8,9,10,11,12,13,14,15,16,17].

Regardless of the language-production modality, the prelinguistic stage refers to the unobservable conceptual apprehension [18,19], conceptual or event representation [19,20], or conceptual organization (CO) [21] in the mind of the speaker or writer, which is subsequently grammatically encoded [4]. Although these terms originate in different theoretical traditions, they all refer to an internal semantic representation of external or internal stimuli. In this manuscript, the term CO is used as an encompassing construct for this prelinguistic semantic organization.

Despite the distinction between prelinguistic and linguistic stages, the structure of the language produced during the linguistic stage can reveal prelinguistic patterns of CO and broader cognitive traits, including abstract reasoning ability [22], working-memory load [23], and individual differences in personality, emotional expression, and mental health [24,25,26]. Advances in computational linguistics and natural-language processing have made it possible to quantify these patterns objectively [27]. One such measure is the analytic thinking score (ATS), developed within the Linguistic Inquiry and Word Count (LIWC) framework [28,29].

The ATS is a continuous variable ranging from 0 to 100 that indexes the relative degree of categorical versus narrative thinking expressed in language. High ATS values are associated with categorical function words, especially articles and prepositions, whereas lower values are associated with more narrative or context-dependent function words, including adverbs, pronouns, negations, auxiliary verbs, and conjunctions. For example, consider the text “The car by the house near the building was in the garage. He drove it there because they felt really scared and could not stay inside anymore.” The first sentence is article- and preposition-dense and therefore receives a high ATS (100), whereas the second sentence contains more narrative/context-dependent function words and therefore receives a low ATS (0.05). The overall ATS (66.02) reflects the relative balance of these linguistic markers.

ATS has been assigned psychometric [25,27] and clinical significance [21,30]. For example, in our previous works [21,30] we showed that individuals with first-episode schizophrenia produced spoken responses with significantly lower ATSs than healthy controls, suggesting that reduced linguistic structure may reflect conceptual disorganization, or lower CO. Notably, this effect emerged despite no group differences in the overall proportion of function and content words, emphasizing ATS as a stylistic marker of thought organization rather than merely a marker of lexical quantity.

Observational studies indicate that ATS differs across spoken and written registers, with higher scores typically observed in written language [29,31]. Moreover, university students whose essays contain a greater proportion of categorical function words relative to narrative function words, that is, a higher ATS, tend to achieve higher academic grades [22]. More recent work also links ATS in written registers to cognitive variables [32]. These findings suggest that writing may provide especially favorable conditions, for example, increased attentional focus for the generation of organized CO. However, the psychological status of ATS has been established primarily through statistical linear models between function-word use and presumed cognitive variables [22,32]. Furthermore, no study has directly compared ATSs across spoken and written language modalities under controlled laboratory conditions while also formalizing the underlying neurocognitive mechanism.

The present work addresses this gap by developing a Bayesian-brain, active-inference model of the relationship between analytic thinking (measured through the ATS) and CO. In our earlier work [21], we proposed a Bayesian-network account in which CO states informed a partially observable Markov decision process (POMDP) architecture; however, the POMDP component was not formally estimated or treated as the central object of analysis. The present manuscript advances that prior work by specifying and estimating an active-inference Markov decision process (MDP) model in which speaking and writing cues are modeled as sensory observations that update beliefs over internal CO states. More specifically, the study’s contribution is the application and formal estimation of the active-inference framework in the context of modality-related differences between writing and speaking.

The paper proceeds in four steps. First, it establishes the idea of precision-weighted inference over CO through a succinct conceptual link between the words-as-attention assumption in natural-language processing, CO, and attention as a synaptic gain mechanism. Second, it formalizes this link at Marr’s [33] three levels of analysis within the complementary frameworks of low-road and high-road active inference [34]. Third, it instantiates the model through a laboratory-controlled experiment. Finally, it assesses a preliminary and model-constrained construct validation of the MDP model within the framework of Variational Laplace, free energy, and Bayesian model selection.

### 1.1. From Words-as-Attention to Attention as Synaptic Gain During Precise Inference of CO

Traditionally, natural-language processing approaches for inferring unobserved cognitive processes from observed fine-grained linguistic features, such as word counts, have assumed that words provide observable evidence of attention. This idea is often described as the words-as-attention assumption [35]. Eye-movement research [35] has also provided evidence for the fundamental role of attention during the unobserved prelinguistic stage of language production, when speakers and writers selectively sample and organize information before encoding it into language. Together, these lines of research suggest that ATSs derived from computational analyses of linguistic registers may reflect attentional foci that emerge during unobservable or hidden prelinguistic CO and are later encoded into observable language.

From a neurobiological perspective, attention can be understood as synaptic gain control. Synaptic gain refers to the responsiveness of neuronal populations to incoming input: when gain is increased, the same sensory input produces a stronger postsynaptic response and has greater influence on downstream processing. In predictive-processing and active-inference accounts, attention is often interpreted as increasing the gain of neuronal populations that encode prediction errors, thereby making selected sensory signals more precise, or more influential, during perceptual inference. Heuristically, focusing attention on a stimulus means giving that stimulus more weight when the brain updates its interpretation of the stimulus content.

The picture-description task provides a suitable experimental design for assessing whether ATS indirectly relates to attention during prelinguistic CO. In this task, participants are cued to either speak or write a narrative about the content of a picture. The central claim is not that writing directly produces a higher ATS. Rather, the speaking or writing cue helps the participant interpret what kind of CO is required for the task. In the formal model introduced below, this cue-guided interpretation is represented as the precision with which (sensory) speaking or writing cues guide inference over (internal) CO. When the cue is treated as clear and reliable, that is, precise, it produces stronger confidence in the corresponding form of CO.

Under this formulation, a high ATS is expected when the participant becomes highly confident in a writing-associated form of CO; that is, a more stable, categorical, and analytically organized way of preparing the picture content for language. Conversely, a low ATS is expected when the participant becomes highly confident in a speaking-associated form of CO, which is assumed to be more narrative and context-dependent. Therefore, the writing-over-speaking advantage in analytic thinking could be interpreted as the behavioral expression measured through ATSs of more precise cue-guided inference over CO, rather than as a direct consequence of writing per se.

The process described above imposes a computational problem for the participant. During picture description, the participant must determine what kind of CO the task requires: a more categorical, writing-associated form of CO or a more narrative, speaking-associated form of CO. This determination cannot be read directly from the picture itself. Rather, it must be inferred by combining the speaking or writing sensory cue, prior expectations about the picture, and incoming sensory information from the picture. The participant must also determine how much weight, or confidence, to assign to the speaking or writing sensory cue when using it to guide CO. In other words, the task requires not only inference over what form of CO is currently relevant, but also inference over the precision with which the sensory cue should guide that CO. In the following section, we formalize the solution to these problems in terms of high- and low-road active inference [34] respectively corresponding to Marr’s levels of analysis [33] applied to the Bayesian brain and free-energy minimization in living systems [36].

### 1.2. The Bayesian Brain and the ATS Through Marr’s Levels of Analysis: A Low-Road to Active Inference in Analytic Thinking

Marr’s computational, algorithmic, and implementational levels of analysis provide a useful framework for linking cognitive theories, computational models, and neural mechanisms [37,38]. The computational level specifies the problem an agent must solve and why that problem matters. In the present case, the problem is to infer the CO state under which a picture-description response is generated. The algorithmic level specifies the procedures by which this inference is performed. The implementational level specifies how the algorithm could be realized neurally.

In the present formulation, the computational level is expressed as Bayesian inference framed within a two-timestep MDP. Here, the participant is modeled as an active-inference agent that infers a hidden CO state from observed speaking and writing cues. Attention enters the model as the precision with which observed cues are mapped onto hidden CO states. The algorithmic level corresponds to marginal message-passing, whereby posterior beliefs are iteratively updated to minimize variational free energy. The implementational level corresponds to a neural process interpretation of the same updates, in which prediction-error signals and precision-weighted synaptic gain provide a plausible cortical implementation. Prediction error therefore bridges levels: it is the formal quantity that drives belief updating and the signal proposed to be encoded by neuronal populations in the neural implementation. Below, we detail all three levels of analysis.

#### 1.2.1. Computational Level of Analysis: Inferring Latent CO States Through Bayes Theorem

Bayesian approaches to brain function, commonly referred to as the Bayesian-brain hypothesis [34,39,40,41,42,43,44,45], propose that the brain does not passively register the world but infers the hidden causes of sensory input. Because sensory evidence is often ambiguous, incomplete, or noisy, perception requires the integration of prior expectations with current evidence. A prior belief refers to what the system expects before new evidence is considered. Sensory evidence refers to the information currently available to the organism. A posterior belief is the updated belief that results from combining prior expectations with sensory evidence. Formally, this updating process can be described as the inversion of a generative model using Bayes’ theorem:

$$P\left(CO|o\right)=\frac{P\left(o|CO\right)P\left(CO\right)}{P\left(o\right)}$$

Here, CO denotes a hidden state, o denotes an observation (i.e., a speaking or writing cue), P(CO) is the prior belief over hidden CO states, and P(o|CO) is the likelihood, that is, the probability of observing a particular speaking or writing cue if a given CO state were present. P(o) is the model evidence, and P(CO|o) is the posterior belief over hidden states after observing the speaking or writing cue. In words, the active-inference agent infers the most likely hidden CO state by combining what it expected before observing the speaking or writing cue with how strongly the observation supports each candidate CO state.

Active inference extends this Bayesian account by specifying how organisms use generative models to perceive and act. In active inference, there are four main families of models: static perception, dynamic perception, dynamic perception with policy selection, and dynamic perception with flexible policy selection [46]. In all four model families, perception corresponds to updating beliefs so that they better explain sensory observations, whereas action corresponds to changing sensory input so that it becomes consistent with expected or preferred states. In this implementation, the model falls within the family of dynamic perception and is specified as a two-timestep MDP. It therefore formalizes how active-inference agents infer CO states from sensory speaking and writing cues, rather than how they select among alternative actions.

**Figure 1:**
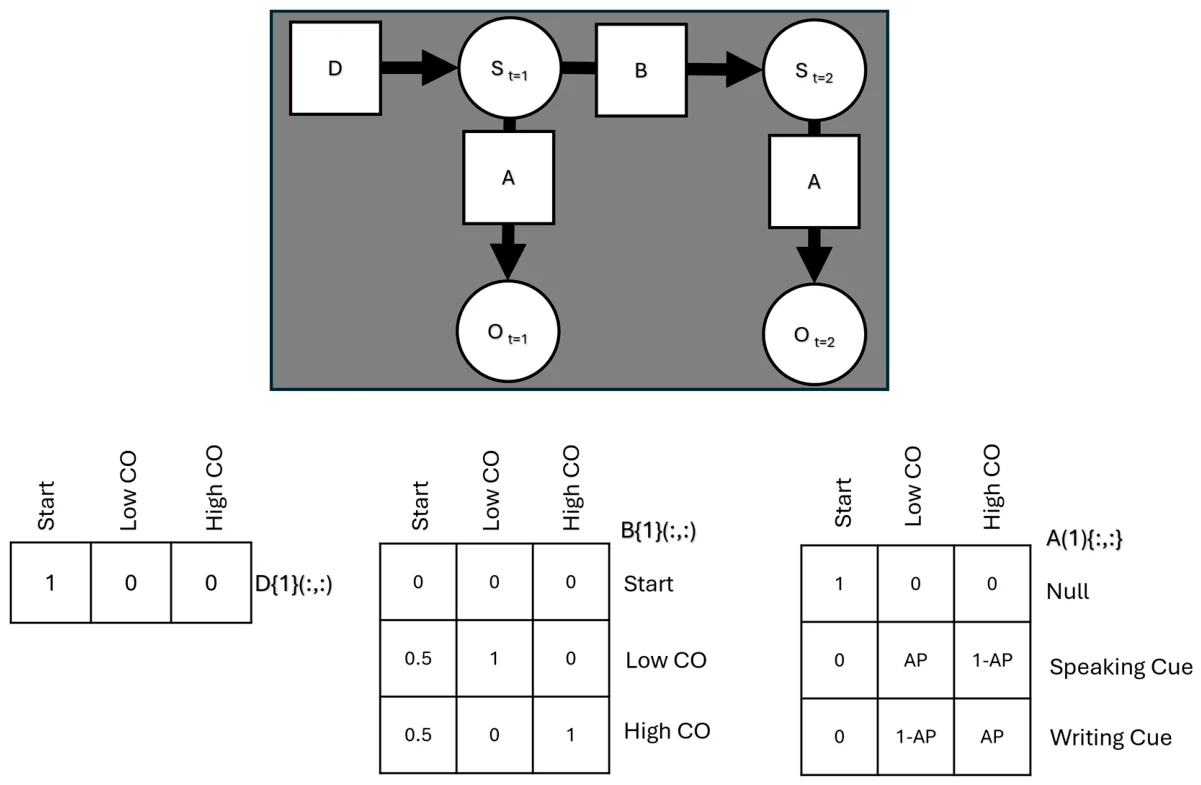
Formal specification of the two-timestep MDP. The D vector fixes the initial state, the B matrix allows transition from the start state to high and low CO states, and the A matrix maps hidden CO states to observed speaking or writing cues. The attention-related parameter AP controls the precision of the cue–CO mapping.

The two-timestep MDP shown in Figure 1 realizes dynamic perception as inference over latent CO states. The hidden-state factor represents the level of CO under which the active-inference agent is preparing to describe the picture. Each trial begins in a start state, after which the agent uses the observed speaking or writing cue to infer whether the current picture-description context is more likely to support low or high CO. The hidden-state space therefore comprises the following states: start, low CO, and high CO.

The observation modality contains three possible outcomes: a start cue, a speaking cue, and a writing cue. The likelihood matrix (A) specifies the probability of each observed cue conditional on each hidden CO state. The D vector fixes prior beliefs over the initial state, whereas the B matrix allows transitions from the start state to either a low-CO or high-CO state.

The likelihood matrix illustrates how the present model adopts the active-inference account of attention as the precision-weighting of sensory evidence. In active inference, attention is treated as the inferred precision, or expected fidelity, of the mapping between hidden states and observations [36,47,48,49,50,51]. In the present model, this principle is applied to CO: the attention-related parameter AP controls the precision and directionality with which latent CO states are mapped onto observed speaking or writing cues.

Formally, the likelihood matrix specifies the probability of observing a start, speaking, or writing cue conditional on the current hidden CO state. The agent inverts this mapping to infer the most probable CO state from the observed speaking or writing cue. When AP = 0.50, speaking and writing cues are equally likely under either CO state, making the mapping maximally ambiguous. Thus, the observed cue provides no differential evidence for whether the agent is in a low-CO or high-CO state. As AP increases above 0.50, the mapping becomes increasingly diagnostic in the hypothesized direction: the speaking cue provides stronger evidence for a low-CO state, whereas the writing cue provides stronger evidence for a high-CO state. Conversely, as AP decreases below 0.50, the mapping becomes increasingly diagnostic in the opposite direction: the speaking cue provides stronger evidence for a high-CO state, whereas the writing cue provides stronger evidence for a low-CO state. In this sense, AP does not represent ATS itself, but the diagnostic precision with which speaking or writing cues disclose the latent CO state that is later expressed linguistically in ATS.

This formulation clarifies how AP relates to ATS. AP does not directly increase ATS. Rather, AP controls the precision and directionality of the mapping between latent CO states and observed speaking or writing cues, thereby determining how strongly an observed cue updates posterior beliefs about the latent CO state. The posterior belief over CO is the immediate computational bridge to ATS. In the present interpretation, high and low ATSs are treated as stochastic behavioral readouts of posterior beliefs over latent CO states: a higher posterior belief in the high-CO state increases the probability of producing discourse that will be measured as a high ATS, whereas a higher posterior belief in the low-CO state increases the probability of producing discourse that will be measured as a low ATS. This can be expressed heuristically as:

P(y_ATS_ = High∣o) ≈ q(s = High CO∣o)

where (y_ATS_) denotes the observed linguistic outcome after ATS measurement, (s) denotes the latent CO state, and (o) denotes the observed speaking or writing cue. This equation should therefore be read as a measurement-level approximation, not as a direct identity between CO and ATS. In the high-road active-inference interpretation introduced below, these low- and high-ATS discourse outcomes are further formally related to AT-low- and AT-high active states.

As posterior confidence in a high-CO state approaches 1, the active-inference agent is assumed to enter an attentional set that supports more stable, categorical, and analytically organized prelinguistic CO, which is expressed downstream as a higher ATS. Conversely, as posterior confidence in a low-CO state approaches 1, the agent is assumed to enter an attentional set that supports less stable or less analytically organized CO, which is expected to yield a lower ATS. The behavioral writing-over-speaking advantage therefore arises when the mapping is precise in the hypothesized direction, such that writing cues provide stronger evidence for high-CO states than speaking cues.

#### 1.2.2. Algorithmic Level of Analysis: Updating Beliefs About CO States Through Marginal Message-Passing

At the algorithmic level, belief updating over CO states is computed through marginal message-passing [52]. The two relevant updating equations can be written as follows:

$$s_{\tau =1}=\sigma \left(\frac{1}{2}\left(lnD+lnB_{\tau }^{†}s_{\tau +1}\right)+lnA^{T}o_{\tau }\right)$$

$$s_{\tau =T}=\sigma \left(\frac{1}{2}\left(lnB_{\tau -1}s_{\tau -1}\right)+lnA^{T}o_{\tau }\right)$$

At the first timestep, posterior beliefs over CO states are updated by combining the prior message from (D), the backward temporal message from the second time point, and the likelihood message supplied by the observed speaking or writing cue. At the second timestep, beliefs are updated by combining the forward message from the first time point with the likelihood message. In both equations, σ denotes the softmax normalization that converts log-probability messages into posterior beliefs over hidden CO states.

The likelihood message ($lnA^{T}o_{\tau }$) is the component of the update through which the observed speaking or writing cue supplies evidence for the latent CO state. The strength and directionality of this evidence are controlled by AP, which determines how precisely latent CO states are mapped onto observed speaking or writing cues in the likelihood matrix. Thus, AP specifically modulates the sensory-evidence term in the message-passing update, while the (D) and (B) terms encode prior and temporal constraints on state inference.

This algorithmic formulation is important because the model contains two distinct time scales. The task itself has two time points, but the inference performed within each time point can involve multiple internal message-passing iterations. Thus, as explained below, a two-timestep MDP can implement an iterative neural updating process while processing a single speaking or writing cue. 

#### 1.2.3. Neural (Implementational) Level of Analysis

In the neural process theory of active inference [47], the brain implements belief updating through a prediction-error formulation of message-passing [46,48]. In predictive-processing formulations, neuronal populations encoding expectations about hidden states generate predictions, whereas prediction-error populations encode the mismatch between incoming evidence and current beliefs. These prediction errors update neuronal activity until posterior beliefs best explain the sensory evidence. Canonical-microcircuit accounts further motivate the interpretation of belief updating in terms of layered cortical message-passing and precision-weighted synaptic gain [49,50].

**Figure 2:**
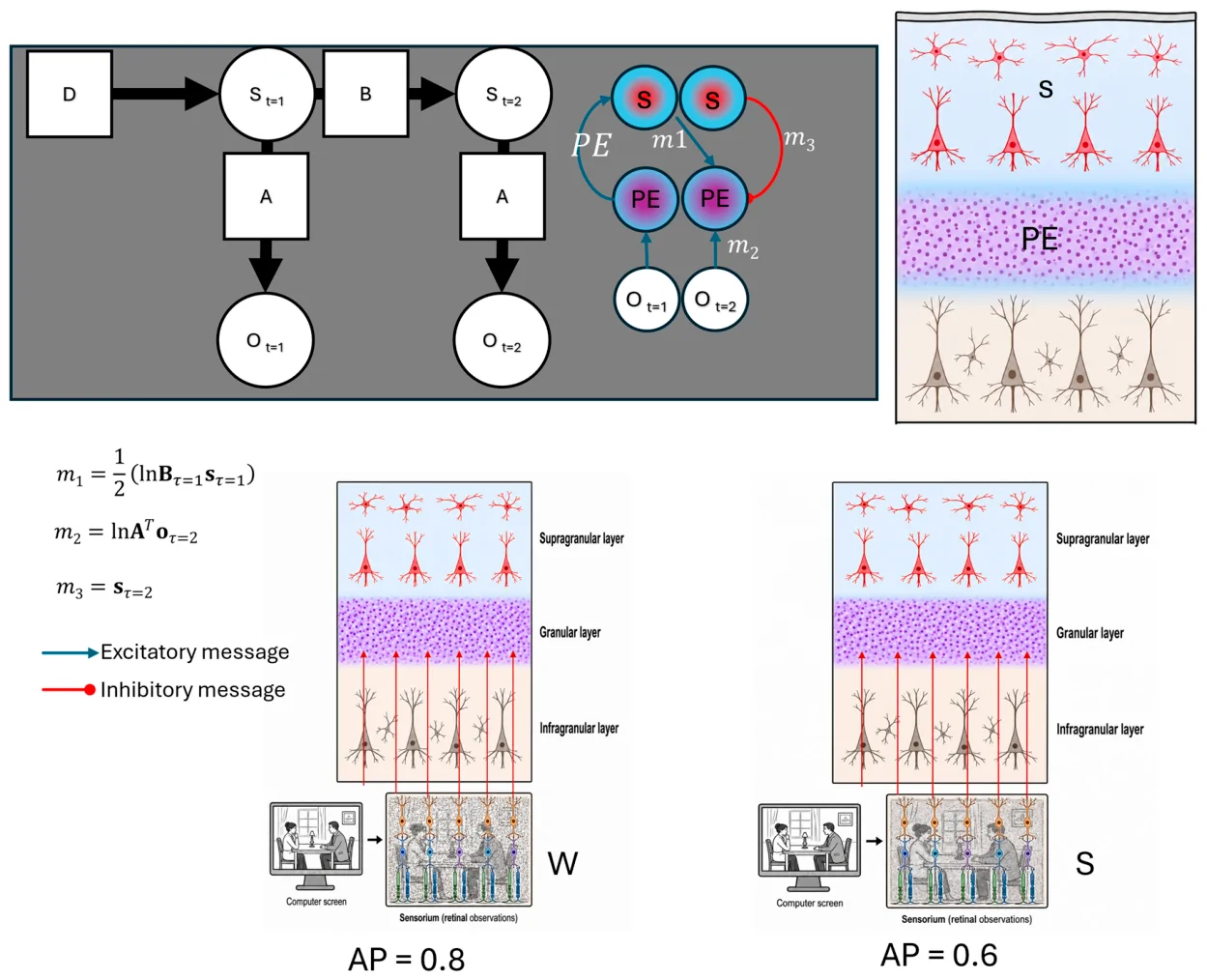
MDP model and prediction-error formulation linking computational, algorithmic, and implementational levels of analysis of the picture-description task. A clear picture-description stimulus and speaking (S) or writing (W) cue are registered as a noisy sensory observation or evidence in the sensorium. Precision-weighted sensory evidence projects to the granular layer and updates beliefs about the hidden CO state through prediction-error message-passing. The comparison between AP = 0.8 and AP = 0.6 illustrates how stronger diagnostic precision in the hypothesized direction produces a less ambiguous sensory message and stronger posterior updating toward the corresponding CO state. In this schematic hypothetical neural implementation, blue arrows represent excitatory messages whereas the red arrow represents the inhibitory message.

Figure 2 depicts the simplified hypothetical neural implementation used in the present model. The computer screen presents a clear picture-description stimulus together with a speaking or writing cue. However, the sensorium registers this input as a noisy sensory observation (i.e., evidence). Within the present formulation, lower diagnostic precision in the cue–CO mapping corresponds to less informative sensory evidence about the latent CO state, whereas higher diagnostic precision corresponds to a less ambiguous sensory message. For example, in the hypothesized direction of the present model, AP = 0.8 provides stronger evidence for the corresponding latent CO state than AP = 0.6. Feedforward sensory evidence projects toward the granular layer, where precision-weighted prediction-error messages are computed. These messages update state representations associated with the inferred CO state in supragranular layers. 

In the picture-description task, prediction-error signals carry evidence about latent CO. A writing cue or speaking cue is compared against predictions generated from current beliefs about the latent CO state. The resulting prediction-error updates neuronal activity encoding posterior beliefs about whether the agent is in a low-CO or high-CO state. AP can therefore be interpreted as a gain parameter on CO-relevant prediction errors. It is important to note that the figure is schematic: it is intended to show how active-inference message-passing can be related to cortical layers, not to assign the entire model to a literal one-to-one anatomical circuit.

For the first and second timesteps respectively, the prediction-error formulation can be expressed as the difference between incoming model-based evidence and the current belief state:

$${PE}_{\tau =1}\leftarrow \frac{1}{2}\left(lnD+lnB_{\tau }^{†}s_{\tau =2}\right)+lnA^{T}o_{\tau =1}-s_{\tau =1}$$

$${PE}_{\tau =2}\leftarrow \frac{1}{2}\left(lnB_{\tau =1}s_{\tau =1}\right)+lnA^{T}o_{\tau =2}-s_{\tau =2}$$

For brevity, we explain the neuronal process at the second timestep (Figure 2). The prediction-error (PE) signal results from the algebraic sum of the agent’s belief about the CO state transition before observing the speaking or writing cue, $\frac{1}{2}\left(lnB_{\tau =1}s_{\tau =1}\right),$ the likelihood message after observing either the writing or speaking cue, $lnA^{T}o_{\tau =2},$ and the current posterior CO state belief, $s_{\tau =2}$. The first two messages are excitatory, and the third message is inhibitory. PE therefore expresses the mismatch between model-based evidence and the agent’s current posterior belief over the CO state. PE then updates the neuronal activity, or membrane voltage, underlying posterior belief. In continuous form, this can be written as:

$$v_{\tau =2}^{\left(k+1\right)}\leftarrow v_{\tau =2}^{\left(k\right)}{\;+\;PE}_{\tau =2}^{\left(k+1\right)}$$

$$s_{\tau =2}^{\left(k+1\right)}=\sigma \left(v_{\tau =2}^{\left(k+1\right)}\right)$$

Here, (v) denotes neuronal activity or membrane voltage, and (k) indexes belief-updating iterations. Importantly, (k) does not denote task time. As introduced above, even in a two-timestep MDP, the system can perform multiple internal neural updates while processing a single observation. After each update, posterior beliefs over CO states are obtained by applying a softmax function to the updated voltage. The iterative process continues until PE is minimized and posterior beliefs settle. At convergence, the posterior belief over CO reflects the hidden organization state that best explains the speaking or writing cue. This posterior belief over CO is then interpreted as the latent inferential state expressed downstream in ATS.

### 1.3. Markov Blanket and Free-Energy Minimization in Living Systems: A High-Road to Active Inference in Analytic Thinking

The preceding model description can be regarded as a low-road active-inference specification [34] because it presents a mechanistic account of how analytic thinking may arise from Bayesian inference over latent CO states. We now present a complementary high-road active-inference interpretation of the same model. High road begins with the idea that a living organism is an adaptive system that must maintain itself within a limited range of viable or preferred states. To do this, it must resist random environmental fluctuations and avoid states that are incompatible with its continued existence. In active inference, this is formalized as surprise minimization [36].

Because surprise is difficult to compute directly, the organism minimizes variational free energy, which is a tractable proxy or bound on surprise. Through perception, the organism updates internal states so that sensory input becomes better explained. Through action, it changes the world or its sensory sampling so that sensory input becomes more consistent with its preferred states.

**Figure 3:**
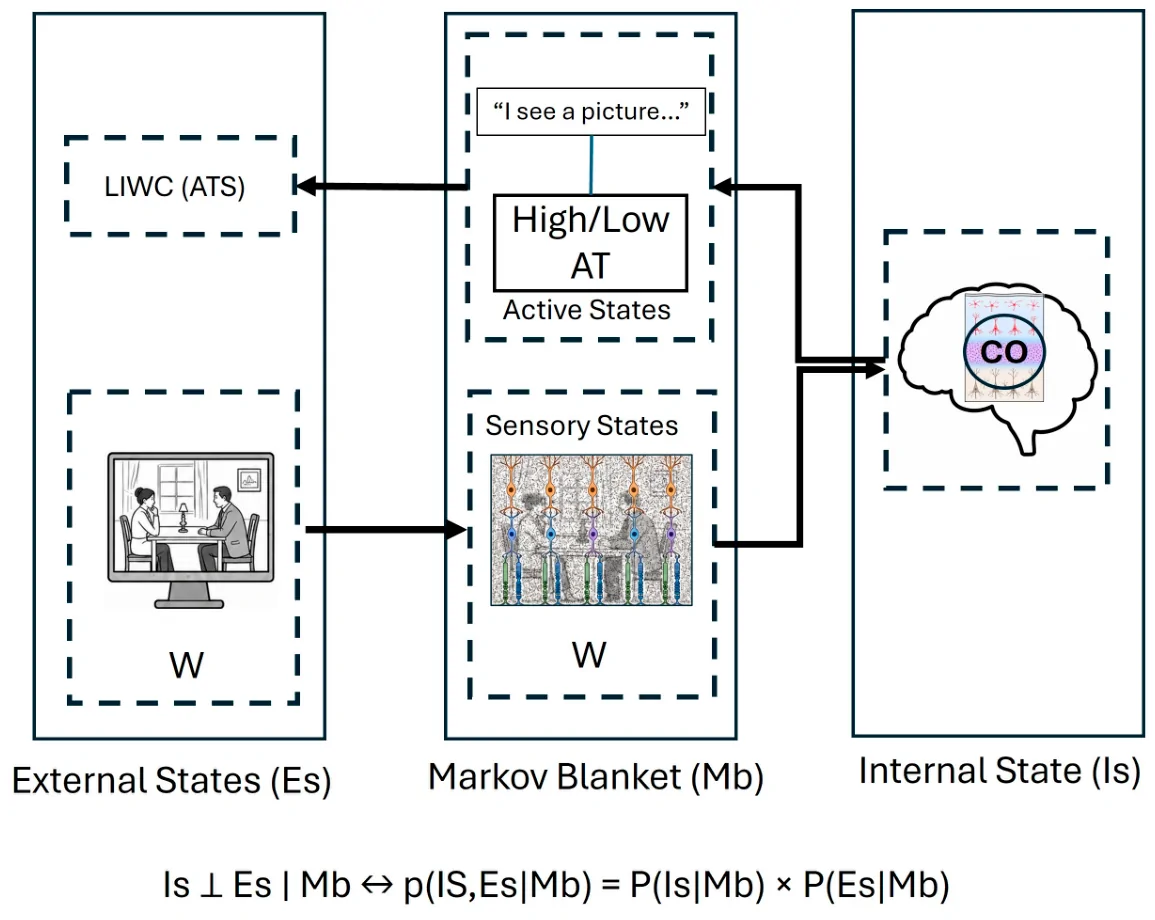
High-road active inference and analytic thinking.

From this perspective, the task can be described in terms of internal states, sensory states, active states, and external states (Figure 3). The internal states correspond to the participant’s inferred CO states. The external states include the task picture, the language-production modality cue, and the observable registers from which the ATS is later computed. Sensory states correspond to the participant’s noisy perceptual registration of the picture and modality cue. Crucially, active states correspond to discourse production through speaking or writing, characterized as relatively low or high in analytic thinking structure, hereafter referred to as AT-low or AT-high active states.

This distinction anticipates the modeling rationale of this work. Participants are not assumed to produce ATS values directly; they produce discourse as realization of discrete AT-low and AT-high active states. ATS is later computed as an external linguistic measure of that discourse. The full rationale and procedure for discretizing ATS as a proxy measure of active states are described in Section 2.

This high-road view assumes that sensory and active states jointly constitute a Markov blanket [40,53]: a statistical boundary that separates the participant’s internal states from the external task state while also mediating their interaction. Crucially, once the sensory and AT active states of the Markov blanket are known, CO states become conditionally independent of external states (i.e., of the ATS). In the present task, this means that the external picture, modality cue, and linguistic product can be related to the participant’s internal CO states only through the sensory and AT active states that mediate perception and discourse production. Conversely, the participant influences the external environment through the AT active states, namely by producing a spoken or written discourse with a particular AT level.

The high-road interpretation therefore draws from the general active-inference claim that the writer or speaker is an adaptive system that must maintain itself within characteristic or preferred internal states by minimizing surprise. In the present model, the participant minimizes variational free energy by updating beliefs about the form of CO required by the task.

Accordingly, ATS should not be interpreted as a direct readout of CO itself. Rather, ATS is an externally computed linguistic trace of the discourse produced by the participant. In high-road terms, the participant’s inferred CO state influences the external linguistic product (the register) indirectly through AT active states, that is, through the production of relatively low- or high-analytic-thinking discourse (cf., more or less categorical linguistic style [22]). ATS is then computed from this external linguistic product as a measurement of the analytic thinking structure expressed in discourse.

### 1.4. Summary of the Present Model

Writing is hypothesized to support a more precise, stable, and analytically organized form of CO, which is deployed as an AT-high active state and becomes externally measurable as a higher ATS. Once the agent infers a low-CO or high-CO state, this posterior belief constrains the attentional set under which picture-derived evidence is sampled, organized, and prepared for linguistic encoding. The agent then produces a discourse sample consistent with that inferred CO state. ATS is subsequently computed from this discourse sample as a noisy linguistic index of the analytic thinking structure expressed in language. The candidate or hypothetical causal chain can therefore be summarized as follows:

Production cue → A(AP) → q(CO state) → AT-low/high active state (discourse production) → ATS measurement

In this chain, A(AP) denotes the likelihood mapping controlled by the attention-related precision parameter. Formally, this likelihood specifies the probability of observing a speaking or writing cue conditional on the latent CO state. During inference, the agent inverts this mapping to estimate the posterior probability of low versus high CO from the observed cue. The term q(CO state) denotes this posterior belief over latent CO states. AT-low/high denotes the modeled active-state level at which the agent produces discourse (during the linguistic stage) consistent with the inferred CO state (during the prelinguistic stage). Finally, ATS is the external linguistic measurement obtained from the function-word profile of that discourse. In the following section, we report a laboratory-controlled experiment that instantiates this formulation.

## 2. Materials and Methods

### 2.1. Participants

A sample of N = 17 (13 females) with a mean age of 20 (SD = 2.08) healthy subjects from Brandon University participated in the study. Fourteen participants (82%) identified English as their first language. Participants were recruited using posters, online advertising and through in-person outreach and received compensation of fifty dollars (gift card) at the end of the experimental session. Participants were required to be registered as an undergraduate or graduate student in any department or program at Brandon University, be at least 18 years of age and with no history of any neurological or mental health disorders. There were no other inclusion criteria. Participants provided written informed consent adhering to the regulations of Brandon University Research Ethics Committee. As described below, sample size estimation was based on sequential analysis and optional stopping [54,55].

### 2.2. Optional Stopping for Sample Size Definition

The stopping rule was prespecified based on two criteria: (1) a Bayes factor greater than 10 for the Bayesian ANCOVA testing the statistical effect of production modality on ATS while controlling for number of words as a covariate of no interest, and (2) a posterior probability greater than 0.95 for the group-level AP parameter in the PEB analysis. These criteria were chosen to ensure strong Bayesian evidence [56] for both the behavioral modality effect and the model-derived parameter effect.

The procedure was implemented in two steps. First, we collected data from an initial sample of 10 participants without conducting interim analyses. After this initial sample was completed, we estimated the Bayesian ANCOVA and the MDP/PEB model at n = 10. At this point, both prespecified evidential criteria were already met: the ANCOVA exceeded the Bayes-factor threshold for the effect of modality, and the PEB analysis exceeded the posterior-probability threshold for the AP parameter.

Although the prespecified thresholds were reached at n = 10, we continued data collection to include at least 50% of the additional eligible participants during the data-collection period, with the goal of assessing whether the evidence remained stable as participants were added. Data collection was finalized at N = 17. This final number included two additional participants who had already signed up for the study before data collection was closed.

### 2.3. Procedure

Participants were seated at a computer desk and provided verbal instructions of the experimental procedure. All participants received the same instructions and were informed the purpose of the study was to collect discourse samples while viewing pictures. Written instructions were provided on the computer screen before beginning two familiarization trials. Specifically, participants were instructed: “In this task, you will be shown a series of images. For each image, we ask that you carefully observe it while at the same time describing what you see. Describe the picture with as much detail as you can. Tell us what you see in the image, describe any elements you wish, and what you think might be happening. At times, you may be prompted to describe the image out loud. At other times, you may be asked to type your response on the computer keyboard. Please provide as much detail as possible in either format. **There is no right or wrong answer**; we are simply interested in your observations and thoughts. You will have a 2 min window to respond to each image. You may stop early if you have no further comments. After the 2 min is up there will be an opportunity to rest before the next image is presented.” The researcher remained in the room for the entire experiment.

### 2.4. Stimuli

Experimental stimuli consisted of ten images taken from the thematic apperception test bank [2] and were presented on a Dell Pro 24 Plus (P2425H) computer monitor positioned approximately 30 inches away from the participant. All pictures were presented at a consistent size (portrait, 5 × 7 inches) and displayed on a neutral gray background. Directly below the image, the language condition cue was indicated using a single capitalized letter (S for speak, W for write) in black type font. The image and language condition cues were presented simultaneously to the participant. Prior to being exposed to the experimental block, participants completed a familiarization trial using two black and white images that did not belong to the thematic apperception test bank and had the opportunity to ask any questions regarding the task before moving onto the experimental stimuli.

### 2.5. Experimental Task

Participants completed an experimental block viewing 10 images, responding to 5 in written modality and 5 in spoken modality, with the opportunity to rest after images. Image order and response modality were randomized and counterbalanced across participants. Participants were simultaneously presented with an image and response modality cue and had 120 s to respond to each image. As specified in the task instructions, participants were requested to describe the image in as much detail as possible and that they could create a narrative or story about what they saw. Spoken responses were audio-recorded using a headset-mounted microphone and were later transcribed by the researcher. Written responses were typed using the keyboard and automatically logged into an excel file on the computer system. The task was built using PsychoPy-2024.2.4 [57].

### 2.6. Data Preprocessing, Analysis, and Modeling

The analytic strategy comprised four stages: data preprocessing, descriptive statistical analysis, MDP modeling, and model-constrained construct-validity analysis.

#### 2.6.1. Data Preprocessing

Preprocessing in the present study was limited to rule-based correction of surface-level textual features before LIWC/ATS extraction. For the spoken condition, non-lexical speech artifacts, such as fillers and stutters, were removed from transcribed speech. For the written condition, an additional parallel preprocessing step was introduced and limited to correcting obvious typos and abbreviations. No semantic rewriting, stylistic editing, or content-level modification was performed in either condition.

Spoken and written responses were processed by one trained rater, who was also a coauthor of the study. The rater was not blind to condition because the modality of each sample was directly evident from the nature of the text and from the preprocessing rule being applied. For example, fillers and stutters occur in spoken transcripts, whereas typos and abbreviations occur in written responses. However, the rater was blind to the experimental hypothesis at the time of preprocessing. All 170 discourse samples were formatted as individual plain text files.

Using LIWC-22 [28], we obtained trial-wise ATS. Because preprocessing could influence a function-word-based measure such as ATS, we added a raw-versus-processed sensitivity analysis (Section 3) in which ATS was obtained from both the original and processed texts. This analysis tested whether preprocessing altered the writing-over-speaking effect on ATS.

#### 2.6.2. Descriptive Statistical Analysis

A general descriptive statistical analysis was conducted to answer whether the writing condition was associated with higher ATS than the speaking condition. As indicated in Section 2.2, we fit a Bayesian ANCOVA model to continuous ATS. Using Bayes factors, we compared a model comprising condition as a fixed effect and subject and trial as random effects against the null model and against a model that included number of words as a covariate of no interest. As elaborated below, the rationale for including this covariate follows from the measurement structure of LIWC-based indices.

LIWC is a word-count-based text-analysis method that estimates psychological and linguistic categories from the distribution of words in a text. ATS is reported as a standardized score. Therefore, response length is a central methodological consideration: shorter texts provide fewer word observations from which to estimate ATS, whereas longer texts provide more stable scores. Consistent with this, we treated number of words as a covariate to ensure that the observed effect of production modality on ATS was not reducible to differences in response length.

A crucial modeling decision in any Bayesian analysis is specifying the appropriate set of priors [46]. Therefore, we added prior-sensitivity analyses. For the Bayesian ANCOVA, we varied the prior coefficient width for the fixed effect of condition and for the effect of the covariate.

#### 2.6.3. MDP Modeling

The central analytic approach of the current work was the MDP analysis of discretized ATS as a proxy measure of AT-high and AT-low active states. This analysis answered a different question from the descriptive statistical analysis: whether speaking and writing sensory cues differentially support inference over internal CO states within a discrete-state active-inference model. To answer this question, ATS values were discretized at the subject level and collapsed across conditions based on each participant’s median distribution. Scores equal to or below the median were categorized as AT-low, whereas scores above the median were categorized as AT-high.

The theoretical rationale for discretization follows from the distinction, made in active-inference formulations, between continuous-state/action models and discrete-state/action models [34,58,59], as well as from the high-road active-inference formalization of the current model explained in the Introduction. Continuous-time model formulations are appropriate for modeling continuously evolving states and movements, whereas discrete-time formulations model inference over discrete hidden states, observations, and policies. Discrete actions, that is, active states, are choices among a finite set of alternatives. Examples include selecting whether to turn left or right in a foraging task, deciding where to look next, or selecting among alternative response options, such as between two discourse-type productions. Continuous actions, by contrast, involve the implementation of movement through continuously evolving dynamics. Examples include eye-movement trajectories, reaching movements, postural regulation, oculomotor control, or handwriting-like motor trajectories.

Participants do not produce ATS values; they produce discourse. In the current active-inference formulation, discourse production is a discrete active state. Therefore, the present model is formulated at the discrete discourse-selection level. The agent is modeled as inferring a latent CO state and then producing an active state consistent with one of two analytic thinking modes. ATS is a post hoc linguistic score derived from the function-word profile of that discourse. Therefore, ATS is treated here as an empirical index and noisy measurement readout of the produced discourse, not as the action itself.

This modeling decision is congruent with our previous active-inference work on grammatical complexity [60]. In that work, grammatical complexity was also an NLP-derived continuous linguistic measure, but the model did not assume that the agent selected a continuously varying grammatical-complexity value. Instead, discourse production was represented as a stochastic selection between low- and high-complexity discourse modes, with the observed linguistic score serving as the measurable output of that latent production process.

This formulation is also consistent with the original conceptual basis of the analytic thinking measure proposed by Pennebaker, Chung, Frazee, Lavergne and Beaver [22]. Specifically, they introduced ATS, referred to as the categorical thinking score, as a function-word-based index reflecting the degree to which language is more categorical versus more dynamic. Thus, although the resulting score is continuous, the construct contrasts between two different thinking and discourse styles. Our discretization therefore operationalizes, via a discrete-state MDP, the contrast between relatively categorical/formal and relatively dynamic/narrative modes of discourse production.

#### 2.6.4. MDP Parameter Estimation and Model Quality Check

At the subject level, we estimated the AP parameter using variational Bayes, specifically the variational Laplace approximation [61]. The planned AP prior was specified in logit space as a Gaussian prior with a mean of 0 and a variance of 0.5. This corresponds to a prior centered at AP = 0.50 on the probability scale. To formally assess whether AP exceeded its non-diagnostic value at the group level, we submitted the first-level posterior estimates to a second-level Parametric Empirical Bayes (PEB) model [62,63]. As prior-sensitivity analysis, we also report parameter estimates and uncertainty intervals under narrower and wider prior variances, in addition to the prior variance used in the planned analysis. Specifically, we compared prior variances of 0.25, 0.50, and 0.75. Relevant second-level PEB analyses are also reported.

We report parameter recoverability, model recoverability, and posterior predictive performance [64]. For parameter recoverability, we simulated data across AP values ranging from 0.05 to 0.95 in increments of 0.05 and then refit the model to recover the generating parameter values. Parameter recovery was quantified using Pearson’s correlation between true and estimated AP values. Model recovery was assessed by fitting the MDP and a variational Laplace linear model to MDP-generated datasets using subject-wise estimated AP parameters. The rationale for this alternative model choice is provided in the Section 2.6.5. Model recoverability was assessed using the difference in free energy. That is, the model with the less negative free energy was deemed the recovered model.

Finally, we performed a posterior predictive check to assess whether behavior generated from the fitted MDP reproduced the empirical condition-level pattern observed in the behavioral analysis. At the subject level, AT-high and AT-low active states were sampled from the posterior over CO states given the modality cue. A generalized linear mixed-effects model with condition as a fixed effect and subject as a random effect was fit to the sampled choices. The results were compared with those obtained from fitting a similar mixed-effects model to the discretized observed ATS.

#### 2.6.5. Model-Constrained Construct-Validity Analysis

We report a preliminary construct-validity analysis constrained by the assumptions of the present model and task. Specifically, we compared the MDP model against a simpler descriptive model of discretized ATS estimated under Variational Laplace. Our model comparison was intended to answer a specific heuristic question relevant to the AP parameter as index of the proposed construct of interest: Does the additional mechanistic structure introduced by the active-inference MDP model improve the explanation of the data sufficiently to justify its greater complexity? If the AP parameter did not add explanatory value, then the simpler descriptive model should provide a better account of the data once model complexity was taken into account. In this sense, the comparison was not intended to prove that the present MDP is the best possible model, but to test whether the AP-based generative model was useful relative to a simpler direct condition-effect account.

This comparison is also statistically motivated by the existing ATS literature, including our own previous work. To our knowledge, previous ATS studies have primarily used simpler descriptive, correlational, or direct predictor-outcome models. These models are simpler than the MDP proposed here because they treat ATS as an observed outcome associated with measured variables; they do not specify hidden CO states, cue-to-state likelihood mappings, Bayesian belief updating, or a precision-like parameter such as AP. 

Variational Laplace [65] provides a common Bayesian estimation framework for the intended comparison because it returns an approximation to the log model evidence, or variational free energy, for each fitted model. This is important because model evidence evaluates accuracy and complexity jointly. A model is favored only when its gain in explanatory accuracy justifies its additional complexity (the core of our heuristic construct-validity question). Therefore, comparing the variational free energies of the linear model and the MDP provides a principled test of whether the mechanistic active-inference model explains the ATS data better than a simpler descriptive account.

At the group level, we used random-effects Bayesian model selection following Rigoux et al. [66]. This framework is appropriate because it does not assume that the same model generated every participant’s data. Instead, it treats model identity as a random effect and estimates the expected frequency with which each model occurs in the population. This is especially suitable for behavioral and cognitive data, where participants may differ in the extent to which their responses are governed by the hypothesized latent mechanism. The protected exceedance probability was used as the main index of model superiority because it estimates the probability that one model is more frequent than the competing model while correcting for the possibility that apparent differences in model frequencies arise by chance. The Bayesian omnibus risk was also reported because it estimates the probability that the apparent model-frequency differences are not meaningful.

For the descriptive/statistical analyses, we used JASP [67]. For modeling, we used SPM12 (http://www.fil.ion.ucl.ac.uk/spm/) and modified scripts available in [46,65,68] to specify and estimate the AP parameter of the two-timestep MDP model described and explained in the previous section.

## 3. Results

### 3.1. Descriptive Statistical Results

**Figure 4:**
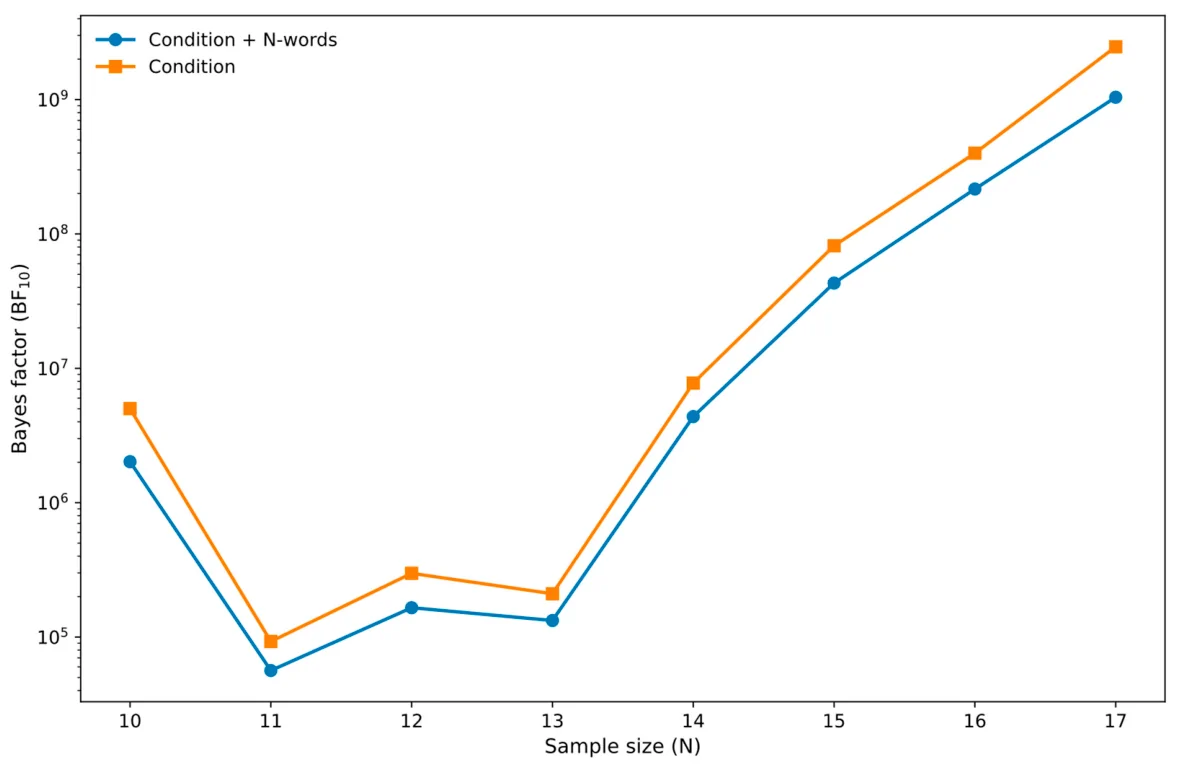
Sequential analysis of the descriptive ANCOVA model. Y axis is set to a base-10 logarithmic scale.

Table 1 summarizes the behavioral results. Mean ATS was higher in the writing condition than in the speaking condition in both the preprocessed and raw datasets. A Bayesian ANCOVA model confirmed this difference and ruled out the effect of number of words. Specifically, the model including the covariate received less support, BF_10_ = 1.168 × 10^9^ for the preprocessed dataset and BF_10_ = 194.82 for the raw dataset, than the model without the covariate, BF_10_ = 2.367 × 10^9^ for the preprocessed dataset and BF_10_ = 436.38 for the raw dataset. The prior-sensitivity analysis (Table 2) and the sequential analysis (Figure 4) respectively showed consistency in the writing-over-speaking superiority effect and no effect of the covariate. Table 3 shows the estimates of the planned ANCOVA model.

### 3.2. Computational Model Results

#### 3.2.1. Model Parameter Estimates and Parametric Empirical Bayes (PEB)

Table 4 shows the subject-level parameter estimates of the MDP model under three different variance priors used for the sensitivity analysis. The subject-level posterior estimates of the attention-related parameter AP, transformed from logit space to probability space, ranged from 0.50 to 0.72. Descriptively, the mean transformed estimate was M = 0.59 (SD = 0.07), indicating that the estimated likelihood mapping was above the non-diagnostic value of 0.50.

**Figure 5:**
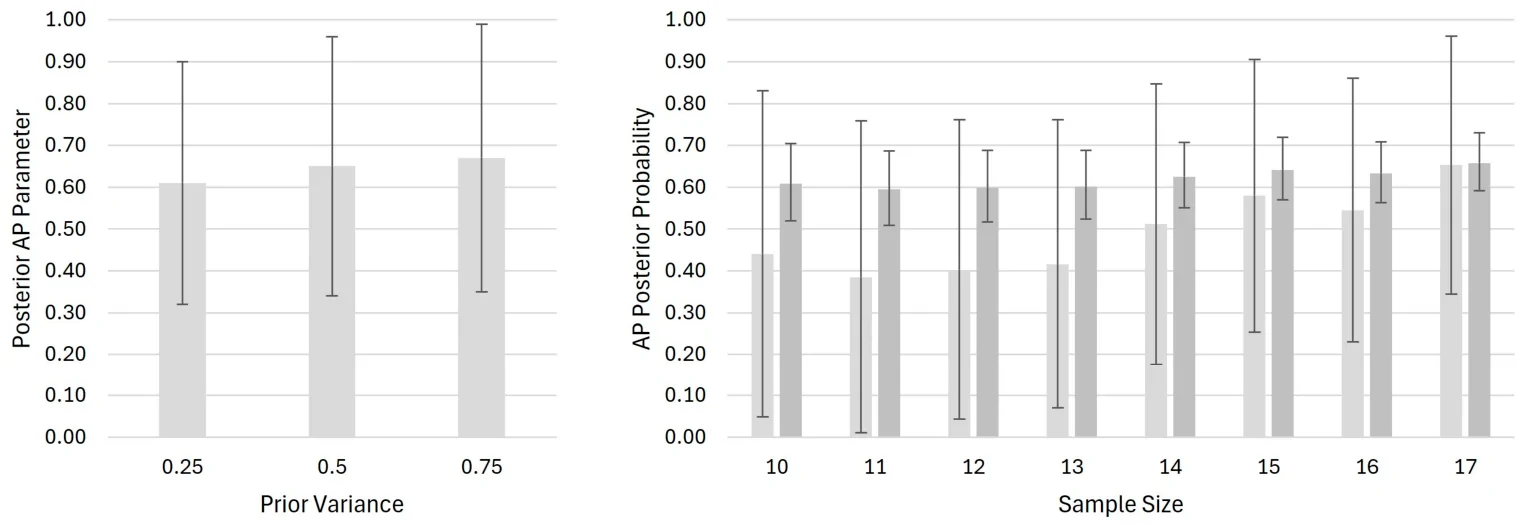
Group-level posterior-mean estimates of the AP parameter. Estimates under three different variance priors (**left**) and under a 0.5 prior variance (**right**, during the optimal stopping procedure) show sequential stability of the estimate. Error bars represent 95% credible intervals. AP was estimated in logit space (light gray) at the first level and transformed into probability space (dark gray) only for interpretation. Therefore, in the PEB model and associated SPM output, the relevant null value is 0, not 0.50. This is because a logit-scale value of 0 corresponds exactly to AP = 0.50 on the probability scale. Thus, testing whether the group-level PEB estimate is greater than 0 in logit space is equivalent to testing whether the transformed AP parameter differs from 0.50 on the probability scale.

Figure 5 shows the results of the group-level PEB model. The model yielded positive group-level posterior estimates, AP = 0.61, 0.66, and 0.67 on the probability scale, for the three variance priors used in the sensitivity analysis. The posterior probability that the group-level logit parameter was greater than 0 surpassed 0.95, providing strong evidence that the estimated attention-related likelihood precision parameter exceeded its non-diagnostic value of 0.50 on the probability scale. In the present model, this indicates that, at the group level, the likelihood mapping was diagnostic in the hypothesized direction, with writing cues providing stronger evidence for high-CO states and speaking cues providing stronger evidence for low-CO states.

#### 3.2.2. Posterior Estimate over CO States: AT-Low and AT-High and Active-State Choices

**Figure 6:**
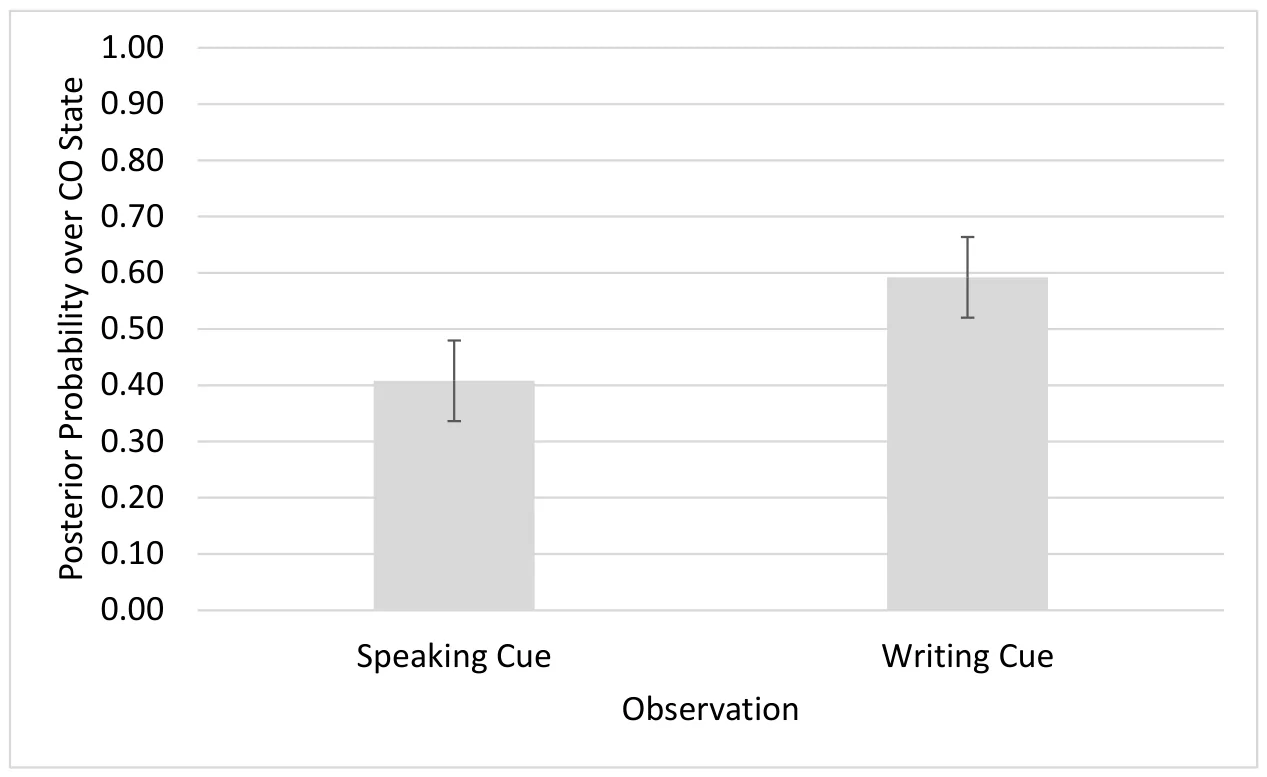
Posterior probability of CO. Error bars represent the standard deviation.

After estimating the AP parameter, we inverted the fitted MDP to obtain posterior beliefs over internal CO states. The cognitive modeling literature refers to this as latent-variable inference analysis [64]. Here, the model is used to infer the internal state that underlies the generation of AT active states. As detailed in the introduction, the artificial agent randomly sampled from this posterior to select the AT active state, that is, the high or low level of analytic thinking with which a text would be produced. For example, after observing a writing cue, the participant is more likely to be in a high-CO than in a low-CO state. Therefore, the agent is more likely to engage in an AT-high active state, producing written discourse with high analytic thinking, which is read out as a high ATS, and vice versa. Figure 6 shows the mean posterior probability of the high-CO state at the group level. The posterior probability was higher in the writing condition (M = 0.59, SD = 0.07) than in the speaking condition (M = 0.41, SD = 0.07).

#### 3.2.3. Parameter and Model Recovery

Table 5 shows the results of the parameter- and model-recovery assessments. Pearson’s correlation between the true and estimated AP parameters was 0.98 (BF_10_ = 1.61 × 10^11^). Crucially, the MDP model was recovered with decisive evidence within the subset of parameter values corresponding to the AP estimates.

#### 3.2.4. Posterior Predictive Check

**Figure 7:**
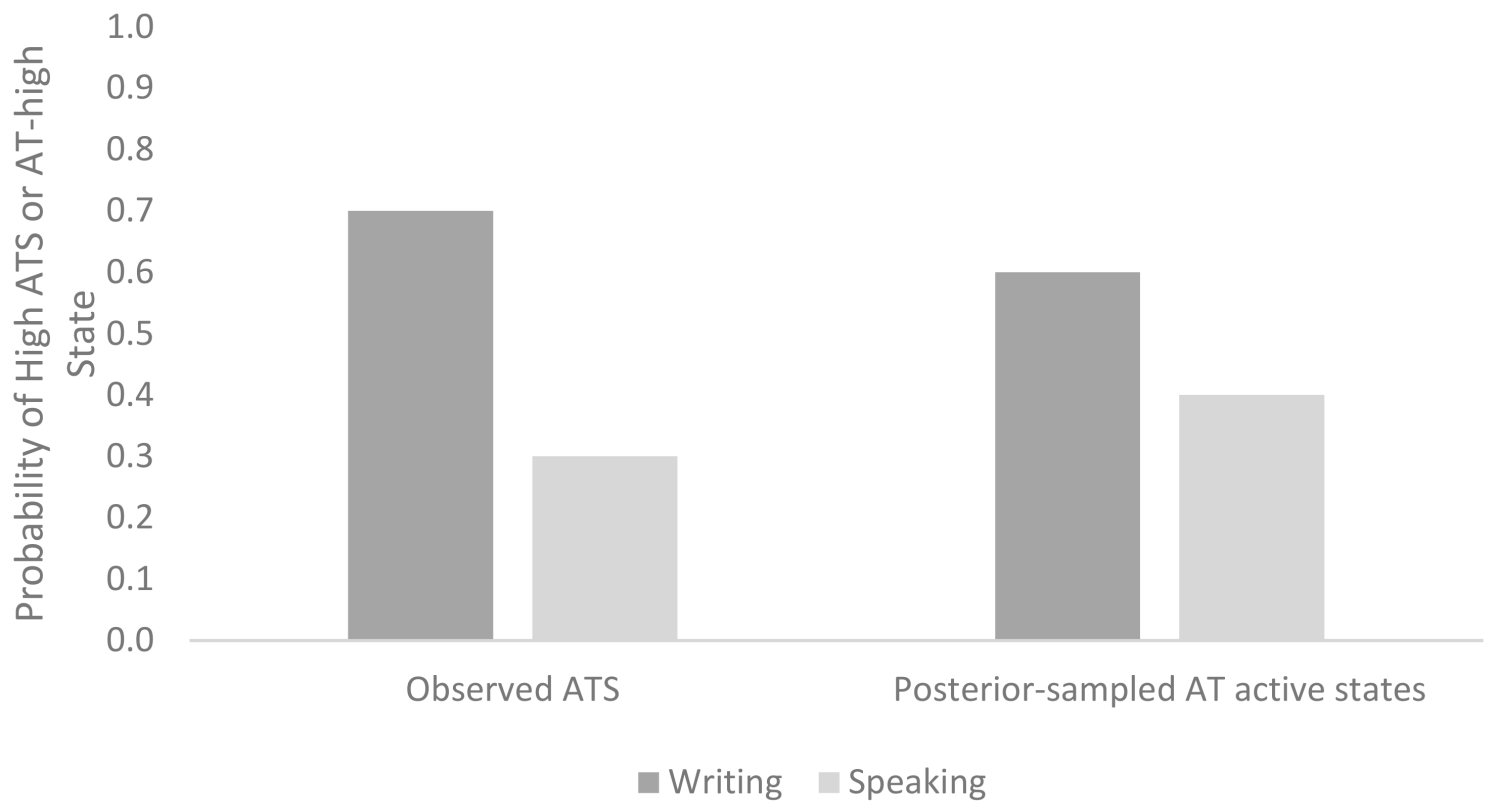
Probability transformation of posterior logit estimates of the Bayesian generalized linear mixed-effects model. Note that high continuous ATS in writing compares to high discretized ATS.

The Bayesian generalized linear mixed-effects model (binomial family) fitted to the discretized observed ATS reproduced the writing-versus-speaking difference obtained with the ANCOVA model of continuous ATS. Table 6 shows the estimates in logit units, and Figure 7 shows the transformed estimates as probabilities. On the probability scale, the observed-data model implied a probability of high ATS of approximately 0.31 in the speaking condition and 0.69 in the writing condition.

As a posterior predictive check, we simulated active-state selection at the individual level and fitted the same generalized linear mixed-effects model to these simulated actions. The model yielded a probability of producing a high-AT active state of 0.40 in the speaking condition and 0.60 in the writing condition (Figure 7). Therefore, this posterior predictive check shows that the artificial agent reproduced the writing-over-speaking advantage observed in the participants’ discretized ATSs. Qualitatively, discretized and continuous ATS are comparable.

### 3.3. Construct Validity: Bayesian Model Selection

Within the domain of a constrained and preliminary construct validation of the current model, random-effects Bayesian model selection favored the active-inference MDP over the Variational Laplace linear model. The MDP showed a protected exceedance probability of 0.95, indicating a high probability that it was the more frequent model in the population after correcting for chance differences in model frequency. The Bayesian omnibus risk was low (BOR = 0.08), indicating a relatively small probability that the observed differences in model frequency reflected random variation. Together, these results indicate that the MDP provided a better account of the ATS data than the Variational Laplace linear model after accounting for model complexity. This finding addresses our heuristic question of whether the additional mechanistic structure introduced by the active-inference MDP, including the AP parameter as the index of the proposed construct of interest, improved the explanation of the data sufficiently to justify its greater complexity.

## 4. Discussion

Focusing on linguistic–behavioral data from a homogenous sample of university students, the present study proposed and tested a preliminary Bayesian-brain active-inference model of the writing-over-speaking advantage in analytic thinking as measured by the ATS. The central claim is that speaking and writing are not merely external production conditions that directly alter analytic thinking; rather, they are observed production cues that provide sensory evidence about latent states of CO. 

Within the model, the observed speaking or writing cue updates posterior beliefs about whether the current picture-description condition is more consistent with a low-CO or high-CO state. These posterior beliefs are hypothesized to establish an attentional set that shapes how picture-derived evidence is sampled, organized, and prepared for linguistic encoding. Accordingly, ATS is interpreted as the downstream linguistic readout (an external state) of AT active states that are shaped by precision-weighted inference over internal CO.

This interpretation is consistent with cognitive-process models of writing, in which writing involves recursive coordination among planning, translating, reviewing, and monitoring processes [8,9,10,11,12,13,14,15,17]. From this perspective, writing may be associated with higher ATS because it permits pausing, rereading, revision, and repeated coordination between the visual stimulus and the emerging linguistic response. Writing also provides a relatively stable external trace of the developing response, which may support the maintenance and reorganization of picture-derived content before it is encoded linguistically. The finding that written responses were associated with higher ATS than spoken responses is therefore interpreted not as a direct effect of writing on word choice, but as the downstream linguistic consequence of a production cue that more strongly supports posterior inference toward a high-CO state. As discussed below, this interpretation extends the words-as-attention assumption by treating observed linguistic markers as traces of cue-guided attentional and inferential processes during prelinguistic CO.

### 4.1. Expanding the Words-as-Attention Assumption to a Formal Active-Inference Framework and Neural Implementation

The present model assigns a formal and biologically, yet preliminary, plausible role to attention, albeit in the specific context of picture-description tasks. The AP parameter determines the diagnosticity and directionality of the likelihood mapping between internal CO states and observed sensory speaking or writing cues. When AP = 0.50, speaking and writing cues are maximally ambiguous because each cue is equally likely under low- and high-CO states. As AP moves away from 0.50, the likelihood mapping becomes increasingly diagnostic. Values above 0.50 indicate the hypothesized mapping, in which writing cues provide stronger evidence for high CO and speaking cues provide stronger evidence for low CO. Values below 0.50 indicate the reversed mapping, in which speaking cues provide stronger evidence for high CO and writing cues provide stronger evidence for low CO.

This distinction is important for interpreting ATS. Greater likelihood precision does not imply that analytic thinking should increase uniformly in both speaking and writing conditions. Rather, precision strengthens posterior confidence in the latent CO state supported by the observed cue. When the likelihood mapping is precise in the hypothesized direction, a writing cue should increase posterior confidence in a high-CO state, which is expected to support more stable, categorical, and analytically organized prelinguistic CO. Conversely, a speaking cue should increase posterior confidence in a low-CO state, which is expected to be associated with lower ATS relative to writing. Thus, precision amplifies the condition-specific consequences of cue-based inference over latent CO rather than exerting a uniform positive effect on analytic thinking.

At the implementational level, this attentional precision can be interpreted as synaptic gain. Specifically, in the prediction-error formulation of the model, the likelihood message expresses what the observed speaking or writing cue implies about the latent CO state. AP determines the precision and directionality of this likelihood message. When AP differs from 0.50, the likelihood message becomes more discriminative, producing a stronger update to neuronal activity or membrane voltage during belief updating. Thus, AP can be interpreted as modulating the strength of the neural message by which the observed speaking or writing cue updates beliefs about latent CO.

The above establishes a possible bridge between the active inference of analytic thinking and Maturana’s biology of language [69]. For Maturana, language is not primarily treated as the transmission of symbolic information, but as an activity of a living system engaged in embodied coordination with its environment. This perspective is consistent with the present model, in which the participant does not simply generate words as isolated linguistic outputs. Rather, the participant observes a picture and modality cue, infers the form of CO required by the task, and produces discourse as an embodied action state. ATS is therefore not interpreted as an autonomous textual property, but as a measurable linguistic trace of the organism’s active state engaged with the task.

### 4.2. From External Validation to a Computational Phenotyping Role of the ATS

ATS has already received evidence for external validity [22,32]. The present account extends this work by providing one possible generative mechanism that could underlie the discourse production (i.e., the AT state) from which ATS is computed. This account also provides initial construct-validation steps. Specifically, the model attempts to connect computational demands, algorithmic belief updating, and plausible neural implementation within a single explanatory framework.

The Bayesian model comparison provides preliminary construct-validating evidence within the evaluated model space for the proposed active-inference account. The linear model captured the descriptive association between production modality and ATS, but it treated speaking and writing as observed predictors acting directly on ATS. By contrast, the MDP embedded this condition effect within a generative architecture in which sensory production cues update posterior beliefs over internal CO states through a precision-weighted likelihood mapping. These internal states then drive AT-high or AT-low active states materialized in more or less categorical discourses from which ATS is computed. Importantly, the MDP-model superiority should be interpreted only relative to the Variational Laplace linear model included in the present two-model comparison.

This initial construct validation also supports the interpretation of subject-level AP estimates beyond classical group-level analysis. Although the group-level PEB estimate indicated that the cue–CO mapping was more diagnostic than the non-informative value of 0.50, individual estimates varied across participants. Some participants showed estimates close to the non-diagnostic boundary, whereas others showed stronger evidence for a precise mapping between latent CO states and observed speaking or writing cues. These differences should not be treated merely as noise around the group mean. Within the active-inference framework, they may reflect meaningful individual variability in the precision with which speaking or writing cues establish posterior beliefs about CO. In psychological terms, some participants may use the speaking/writing cue as a strong organizer of attention, whereas others may rely less strongly on that cue when sampling and organizing picture-derived evidence.

The subject-level AP estimates also have a possible neural, yet hypothetical, interpretation. Participants with AP estimates close to 0.50 would correspond to weaker gain on cue-related prediction-error signals. Values above 0.50 indicate stronger gain in the hypothesized direction, whereas values below 0.50 would indicate stronger gain in the reversed direction. Although no neural data were collected, these differences provide a principled computational hypothesis for future studies: individuals may differ in the degree to which production cues modulate neural gain and thereby organize prelinguistic attentional sampling.

### 4.3. Future Directions and Limitations

The neural interpretation of the current model is hypothetical. Therefore, it opens new lines of research on the generative mechanisms of analytic thinking and its relevant measurement through ATS. Future studies combining the present task with electroencephalography, pupillometry, eye tracking, electrodermal conductance, or functional near-infrared spectroscopy could test this interpretation more directly by examining whether AP estimates farther from 0.50 are associated with stronger cue-evoked signatures of precision-weighted belief updating. In the hypothesized direction, this would mean testing whether AP estimates farther above 0.50 are associated with stronger evidence that writing cues support high-CO states and speaking cues support low-CO states.

For example, the eye-tracking literature on language production provides an empirical bridge between this computational claim and the psycholinguistic process of message formulation. Griffin and Bock [70] showed that speakers’ eye movements during scene description are closely coordinated with sentence formulation and that early fixations reflect rapid apprehension of response-relevant event structure rather than simple capture by visually salient objects. Gleitman, January, Nappa and Trueswell [18] further showed that event apprehension and utterance formulation interact dynamically, challenging a strictly serial view in which thought is fully formed before speech begins. Konopka [3] extends this point by showing that speakers encode not only individual objects but also relational event structure before and during grammatical formulation. Together, these findings support the assumption that language production begins with selective sampling of visual information relevant to the message to be produced.

The robustness and generalizability of the results are limited to comparable participants and comparable experimental conditions. The results, however, suggest that this model could be especially relevant for the study of conceptual disorganization in psychiatry samples. Prior work has shown that individuals with schizophrenia, especially those with clinical symptoms of conceptual disorganization, tend to produce spoken language with a lower ATS compared with healthy controls [21,30]. The present framework suggests that such reductions may reflect disturbances in the precision-weighting of cues or evidence that normally support CO. If the active-inference agent fails to assign sufficient precision to features that support high-CO states, discourse may become less coherent, less structured, and less categorically organized. This interpretation is consistent with active-inference accounts proposing that abnormal perception and cognition may reflect altered precision weighting [34,39,40,41,42,43,44,45,46,48,51]. The current results therefore motivate future studies asking whether writing could partially stabilize CO in affected populations by providing additional opportunities for visual monitoring, self-correction, and resampling of contextual evidence.

Both the reliability of subject-level estimates and initial construct validity are especially important for interpreting ATS as a potential readout of a computational phenotype component. A robust group-level writing-over-speaking effect does not necessarily imply that the same effect is equally strong, equally stable, or equally meaningful for every participant. This issue is central to the reliability paradox [71]: experimental effects may be robust at the group level while showing limited test–retest reliability as individual-difference measures. Different ATS values may therefore be sensitive to trial content, narrative style, typing speed, speech fluency, language background, or idiosyncratic response strategies. Model-derived parameters provide one possible route for addressing this problem because they attempt to estimate the latent mechanism proposed to generate the observed effect.

If future studies show that AP is recoverable, stable across repeated testing, and associated with theoretically relevant external measures, then it could be treated as a candidate computational phenotype component of precision-weighted CO [72]. Such a phenotype component would not identify analytic thinking itself, nor would it reduce CO to a single linguistic score. Rather, it would quantify one computational condition under which analytic linguistic structure emerges: the precision and directionality with which production cues update posterior beliefs about internal CO.

Several limitations should be acknowledged. First, the current model formalizes inference over latent CO states from speaking and writing cues, but it does not model the full process by which CO is constructed over time. CO is represented as a latent state inferred from the cue structure of the task and noisily expressed downstream in ATS, rather than being directly observed or decomposed into its cognitive subcomponents. Thus, the model captures one computational condition under which more or less organized conceptual structure may be expressed in language, but it does not provide a complete model of CO itself.

Second, this study treated the writing cue as a formal manipulation indexing the experimentally cued writing condition rather than as evidence that writing represents a single, isolated process. Although the behavioral design demonstrates a writing-over-speaking advantage in analytic thinking as measured by ATS, it does not identify which specific components of writing drive this effect. Writing differs from speaking in several dimensions, including motor demands, temporal pacing, visual monitoring and feedback, opportunities for pausing and revision, and social–pragmatic context. Any of these components may contribute to the computed ATS. Future research should isolate these factors using approaches such as no-revision writing paradigms, time-matched writing conditions, keystroke logging, pause analysis, and eye tracking to determine whether the advantage is driven by visual monitoring, revision, slower production pace, reduced social pressure, or a combination of these factors. Moreover, although the preprocessed and raw data showed the same relative between-condition difference in ATS, preprocessing had a substantially greater effect on spoken than on written registers. Specifically, the mean word-count difference between raw and preprocessed data was 17.42 words for spoken registers but only 0.37 words for written registers, while the corresponding mean ATS differences were 14.19 and 0.75 points, respectively. A length-matched analysis might partially address this imbalance; however, we did not conduct such an analysis because selecting matched subsets of trials could introduce additional selection bias.

Third, the MDP model presented in this work falls within the category of dynamic perception or predictive coding. Although this is a special case of active inference, it does not include policy selection or expected free-energy minimization. A more complete active-inference model could incorporate active sampling, gaze allocation, revision policies, and the expected epistemic value of different production strategies. Furthermore, a continuous ATS likelihood model is worth including in the model space. Such extensions would allow future models to move beyond cue-based inference over CO states and toward a fuller account of how participants actively sample, monitor, and revise picture-derived conceptual content during speaking and writing.

## 5. Conclusions

The current preliminary evidence obtained in laboratory conditions is consistent with a candidate active-inference account in which writing is associated with a higher ATS by providing sensory cues that support precision-weighted inference toward high-CO states and the ensued AT-high active states. ATS is therefore interpreted as a downstream, externally computed linguistic trace of analytic thinking: it is directly determined by AT active states and indirectly shaped by latent CO states inferred during language production.

## Figures and Tables

**Table 1:** Descriptive statistics of LIWC analysis.

		Preprocessed				Raw			
	Condition	Mean	SD	Min Max		Mean	SD	Min Max	
ATS	Speaking	52.03	28.77	4.6	99	66.19	21.22	25.24	99
	Writing	78.99	22.1	6.2	99	79.32	22.36	2.68	99
Number of Words	Speaking	97.75	55.83	7	216	115.17	56.86	15	224
	Writing	56.97	19.44	8	97	57.34	23.10	8	177

**Table 2:** Prior sensitivity analysis.

Condition Prior	N Words Prior	Random Subject, Trial Prior	Covariate Model	Condition-Only Model
0.25	0.25	1	8.626 × 10^8^	1.601 × 10^9^
0.5	0.354	1	1.168 × 10^9^	2.367 × 10^9^
1	1	1	4.594 × 10^8^	2.533 × 10^9^

Note. Prior sensitivity analysis was performed on the preprocessed dataset.

**Table 3:** Parameter estimates of the planned Bayesian ANCOVA model.

			95% Credible Interval	
Parameter	Mean	SD	Lower	Upper
Intercept	65.55	7.50	57.98	80.44
Speaking	−13.12	1.81	−16.80	−9.62
Writing	13.12	1.81	9.42	16.68

**Table 4:** Parameter estimates of the MDP model.

Subject	AP (0.25)	AP (0.5)	AP (0.75)
1	0.61	0.66	0.69
2	0.52	0.53	0.53
3	0.52	0.53	0.53
4	0.57	0.61	0.63
5	0.50	0.50	0.50
6	0.52	0.53	0.53
7	0.61	0.66	0.69
8	0.57	0.61	0.63
9	0.61	0.66	0.69
10	0.61	0.66	0.69
11	0.52	0.53	0.53
12	0.50	0.50	0.50
13	0.57	0.61	0.63
14	0.50	0.50	0.50
15	0.57	0.61	0.63
16	0.65	0.72	0.75
17	0.61	0.66	0.69

**Table 5:** Parameter and model-recovery assessment.

True AP	Estimated AP	MDP-F	Linear-F	MDP-F Minus Linear-F	Recovered Model	BF	Evidence
0.05	0.09	−24.94	11.33	−36.27	Linear	0.00	No preference
0.10	0.14	−37.55	−31.54	−6.01	Linear	0.00	No preference
0.15	0.16	−42.81	−43.26	0.44	MDP	1.56	Weak favor
0.20	0.28	−59.51	−70.17	10.66	MDP	42,555.97	Decisive
0.25	0.24	−55.31	−63.33	8.03	MDP	3059.29	Decisive
0.30	0.39	−67.90	−79.59	11.69	MDP	119,893.28	Decisive
0.35	0.33	−64.46	−76.30	11.84	MDP	138,657.63	Decisive
0.40	0.43	−69.41	−82.01	12.60	MDP	296,771.30	Decisive
0.45	0.46	−72.37	−83.08	10.72	MDP	45,062.36	Decisive
0.50	0.54	−70.31	−82.97	12.66	MDP	315,182.90	Decisive
0.55	0.55	−70.14	−82.83	12.69	MDP	323,764.20	Decisive
0.60	0.69	−62.98	−74.32	11.34	MDP	83,891.47	Decisive
0.65	0.56	−69.93	−82.62	12.69	MDP	323,485.55	Decisive
0.70	0.76	−55.31	−63.63	8.32	MDP	4108.52	Decisive
0.75	0.80	−48.93	−52.33	3.40	MDP	29.99	Decisive
0.80	0.76	−55.31	−63.61	8.30	MDP	4035.43	Decisive
0.85	0.84	−42.81	−42.88	0.07	MDP	1.07	Weak favor
0.90	0.86	−39.38	−35.58	−3.80	Linear	0.02	No preference
0.95	0.91	−24.94	11.77	−36.71	Linear	0.00	No preference

Note. Model recovery was assessed using variational free energy (F). A positive MDP-F minus Linear-F value indicates that the MDP explained the MDP-generated data better than the Variational Laplace linear model. The Bayes factor (BF) provides an equivalent interpretation of the free-energy difference [61]. Specifically, BF was computed as exp(MDP-F minus Linear-F), that is, BF_12_ ≈ exp(F_1_ − F_2_), where F approximates log model evidence [61]. Gray-shaded rows show the range of the recovered model including the subset of parameter values relevant to the estimated AP parameters.

**Table 6:** Parameter estimates of the Bayesian generalized linear mixed-effects model of active states.

			95% Credible Interval				95% Credible Interval	
	Estimate	SE	Lower	Upper	Estimate	SE	Lower	Upper
Intercept	0.01	0.18	−0.33	0.38	−0.01	0.06	−0.15	0.12
Speaking	−0.81	0.19	−1.26	−0.44	−0.38	0.07	−0.54	−0.23
Writing	0.81	0.19	0.44	1.26	0.38	0.07	0.23	0.54

## Data Availability

Modeling scripts and raw data files can be downloaded from the Writing-brain laboratory website https://people.brandonu.ca/limongir/home/the-writing-brain-laboratory/.
